# Editorial: Molecular mechanisms of neuropsychiatric diseases

**DOI:** 10.3389/fnmol.2022.1102296

**Published:** 2022-12-08

**Authors:** Mónica Moreira-Rodrigues, Melanie J. Grubisha

**Affiliations:** ^1^Laboratory of General Physiology and Center for Drug Discovery and Innovative Medicines (MedInUP), Department of Immuno-physiology and Pharmacology, School of Medicine and Biomedical Sciences (ICBAS), University of Porto (UP), Porto, Portugal; ^2^Department of Psychiatry, University of Pittsburgh, Pittsburgh, PA, United States

**Keywords:** neuropsychiatric disorders, molecular mechanisms, depression, schizophrenia, post-traumatic stress disorder, intellectual development disorder, catatonia

Neuropsychiatric disorders, such as depression, schizophrenia, bipolar disorder, obsessive compulsive disorder, post-traumatic stress disorder (PTSD), autism spectrum disorder and others are estimated to impair millions of individuals globally. Despite the high global prevalence, much remains unknown about the underlying molecular mechanisms that lead to these and other neuropsychiatric diseases. The likelihood of developing these disorders may depend on a variety of genetic, developmental and environmental factors. The molecular alterations produced by these factors may contribute to both disease onset or the persistence of a specific pathology. Identifying the molecular mechanisms of a disease will increase our understanding of the underlying pathophysiology and ultimately lead to the rational design of targeted treatments. The goal of this Research Topic was to explore fundamental molecular mechanisms underlying neuropsychiatric diseases, and how these mechanisms may be exploited for potential therapeutic benefit.

A longstanding challenge in the elucidation of molecular mechanisms underlying neuropsychiatric disease has been the initial paucity of anatomical and/or molecular findings. Distinguished American neurologist Dr. Fred Plum once referred to schizophrenia as “the graveyard of neuropathologists.” This Topic features multiple papers that serve to tackle this and other challenges and link clinical entities to anatomical and molecular abnormalities.

Ren et al. showed that schizophrenia patients with auditory verbal hallucinations exhibit a decrease in cortical thickness in orbitofrontal cortices, which was negatively correlated with severity of these symptoms. Seeking to discover molecular aberrations in a depression-relevant model, Chen et al. employed a large-scale-omics approach to achieve metabolic profiling in the olfactory bulb of a chronic mild stress mouse model. They discovered an abnormal metabolism of the tryptophan pathway in the olfactory bulb, which may mediate the occurrence of a depression-like phenotype in a chronic mild stress model. On the other way, Martinho, Oliveira et al. sought to identify specific molecular abnormalities underlying contextual fear memory relevant to PTSD, and that modulating this system can reverse fear-related phenotypic changes. In Martinho, Oliveira et al., they found that epinephrine may be involved in the persistence of traumatic memories in PTSD, possibly through enhancement of the expression of *Nr4a2* and *Nr4a3* genes in the hippocampus. The persistence of contextual traumatic memories may contribute to anxiety-like behavior and resistance of traumatic memory extinction in a PTSD rodent model. In a follow-up study, Martinho, Correia et al. showed that nepicastat, a highly potent central and peripheral DBH (dopamine beta-hydroxylase) inhibitor that is effective in modulating the sympathetic nervous system, decreases the persistence of traumatic memories and anxiety-like behavior in this model, which may have direct translational potential in the treatment of PTSD.

Linking anatomical and phenotypic findings to genetic underpinnings has proved to be another challenge, yet one the field has fearlessly embraced. Although the high complexity of neuronal and cortical structure is regulated by a myriad of genes, Parnell et al. devised a clever strategy to reduce the genetic complexity to a single mutation in a relevant gene to better understand its specific contribution to both normal and abnormal neuronal architecture. Using the E1577K point mutation in Kalirin-7, Parnell et al. showed that in contrast to wild type Kalirin-7, the E1577K mutant failed to drive dendritic arborization, spine density, and NMDAR activity within spines, alongside a robust reduction in Kalirin-7 RAC1 guanine exchange factor activity. Similarly, Che et al. identified a splicing variant and a novel frameshift variant of the BCL11B gene, thus suggesting that an aberrant translation of this gene may lead to an intellectual development disorder. Fujii et al. used a FRET-based approach to understand the impact of CAMK2A (Ca^2+^/calmodulin dependent protein kinase II alpha) gene mutation in patients' phenotype. Han et al. discovered a novel missense single nucleotide polymorphism, rs61753730 (Q152E), located in the fourth exon of the frizzled class receptor 6 gene (FZD6), which they demonstrate impacts depressive-like symptoms of Fzd6-knockin mice. These findings contribute to our understanding of the genetic underpinnings of both normal and abnormal neurodevelopment, thus paving the way for future targeted therapeutics.

Finally, it is imperative that we continue to integrate the field's existing cellular and molecular knowledge with clinical observations. In this Topic, Ariza-Salamanca et al. highlighted molecular and cellular mechanisms leading to catatonia, which had previously been described as a purely clinical syndrome. In a similar vein, Grubisha et al. reviewed post-translational modifications in human post-mortem brain tissues of neuropsychiatric diseases. Translational momentum is fueled by this bench-to-bedside viewpoint, with one constantly informing and advancing the other.

In conclusion, through multiple innovative approaches, this Research Topic advanced our knowledge in understanding molecular mechanisms underlying several neuropsychiatric disorders. The papers herein integrate anatomical, cellular, molecular, and genetic findings in work that will ultimately advance the field toward elucidation of novel therapeutic targets and inform future rational drug design.

## Author contributions

All authors listed have made a substantial, direct, and intellectual contribution to the work and approved it for publication.

